# Cancer-associated fibroblasts in the lung cancer microenvironment from multi-dimensional mechanisms to therapeutic strategies

**DOI:** 10.3389/fonc.2026.1866753

**Published:** 2026-07-22

**Authors:** Yikun Feng, Le Gu, Zhengao Jia, Chunxiao Zhang, Zhengang Zhu

**Affiliations:** 1 First Teaching Hospital of Tianjin University of Traditional Chinese Medicine, Tianjin, China; 2 National Clinical Research Center for Chinese Medicine, Tianjin, China

**Keywords:** cancer-associated fibroblasts, extracellular matrix, immune evasion, lung cancer, therapeutic resistance

## Abstract

**Background:**

Lung cancer remains the leading cause of cancer-related mortality worldwide, and therapeutic outcomes are frequently compromised by drug resistance and the complex tumor microenvironment. Although malignant progression is fundamentally driven by genetic alterations, cancer-associated fibroblasts (CAFs) have emerged as critical regulators of tumor progression, immune evasion, and therapeutic resistance.

**Main body:**

This review systematically summarizes the multidimensional roles of CAFs in lung cancer pathogenesis. CAFs remodel the extracellular matrix to generate high-stiffness stromal barriers, thereby activating the Integrin/Focal adhesion kinase (FAK)/Yes-associated protein (YAP) mechanotransduction axis and impairing drug delivery. Through a multifaceted secretome that includes Interleukin-6 (IL-6), Transforming growth factor-beta (TGF-β), Hepatocyte growth factor (HGF), and C-X-C motif chemokine ligand 12 (CXCL12), CAFs promote epithelial–mesenchymal transition and non-cell-autonomous resistance. Recent single-cell and spatial transcriptomic studies have further revealed functionally distinct CAF subpopulations associated with matrix remodeling, immune exclusion, and therapeutic response. In addition, CAF-derived small extracellular vesicles (sEVs) mediate bidirectional communication with tumor and immune cells, reinforcing tumor plasticity and immune evasion. CAFs also contribute to T-cell exclusion and suppressive myeloid-cell recruitment, thereby attenuating the efficacy of immune checkpoint blockade. Finally, we critically evaluate emerging therapeutic strategies targeting CAF-mediated pathways and discuss their translational opportunities and limitations.

**Conclusions:**

Overcoming CAF-mediated resistance requires a shift from non-selective stromal depletion toward biomarker-guided stromal normalization and subtype-specific interventions. By integrating mechanistic and translational evidence, this review provides a comprehensive framework for developing more effective precision therapies in lung cancer.

## 1 Introduction

Lung cancer, primarily arising from pulmonary tissues, bronchi, or airway epithelium, is a formidable malignancy driven by the cumulative effects of chronic multifactorial carcinogenic exposure, inducing DNA damage and precipitating aberrations in critical molecular signaling pathways (1). Established risk factors include smoking, environmental pollutants, and occupational exposure to carcinogens (1–5). Additionally, chronic lung diseases, family history, and prior chest radiotherapy further elevate the risk of lung cancer (2). According to authoritative data from the World Health Organization (WHO) and the International Agency for Research on Cancer (IARC), smoking remains the leading preventable cause, implicated in approximately 85% of cases (1, 6). In the United States, smoking is associated with 80%–90% of lung cancer deaths, with smokers facing a 15- to 30-fold higher risk than non-smokers (7). Global projections indicate that the burden will intensify, with an estimated 4.62 million cases and 3.55 million deaths by 2050, reflecting a persistent trend of high incidence and mortality (8–11).

The pathogenesis and progression of lung cancer are driven not only by cell-intrinsic genetic alterations but are also shaped by stromal and immune components within the tumor microenvironment (TME) (12, 13). Cancer-associated fibroblasts (CAFs) represent one of the predominant and phenotypically plastic cell population within the solid tumor stroma, where they remain persistently activated in close spatial proximity to malignant nests (14). CAFs primarily originate from resident fibroblasts. They may also arise from epithelial cells through epithelial–mesenchymal transition, endothelial cells through endothelial–mesenchymal transition, mesenchymal stromal cells, and, in certain contexts, adipocyte-derived or bone marrow-derived stromal cells. In some contexts, adipocyte-derived and bone marrow-derived stromal cells may further contribute to their origins (15, 16). Characterization via transcriptomic and functional profiling has identified distinct CAF subpopulations, notably myofibroblastic CAFs (myCAFs) driven by the Transforming growth factor-beta (TGF-β)/Suppressor of mothers against decapentaplegic (SMAD) signaling pathway, inflammatory cancer-associated fibroblasts (iCAFs) modulated by the Interleukin-1 (IL-1)/Janus kinase/signal transducer and activator of transcription (JAK/STAT) axis, and antigen-presenting CAFs (apCAFs) with potential immunomodulatory roles (17). From a functional perspective, CAFs induce matrix stiffening by augmenting extracellular matrix (ECM) deposition and contractility. This process activates Yes-associated protein (YAP)-dependent mechanotransduction pathways, which subsequently facilitate tumor cell migration and invasion (18). Concurrently, the rigid stromal architectures established by CAFs serve as physical barriers to immune cell infiltration. By secreting bioactive factors that recruit suppressive immune populations, CAFs foster a state of immune exclusion that substantially compromises the efficacy of immunotherapeutic interventions (19–21). Furthermore, CAF-mediated stromal remodeling elevates interstitial fluid pressure (IFP), which subsequently compresses microvessels and impedes the effective diffusion of therapeutic agents, thereby systematically driving therapeutic recalcitrance (22–24).

Despite the rapid advancements in CAF research, their biological underpinnings and clinical implications in lung cancer remain characterized by significant controversies and unresolved enigmas. The absence of definitive, lineage-specific markers, coupled with profound functional heterogeneity, has historically led to disparate cellular definitions and analytical scales across various studies. Consequently, conflicting conclusions regarding their pro-tumorigenic or tumor-suppressive roles persist across different experimental models (25). Furthermore, the intricate spatial coupling between distinct CAF subpopulations—encompassing myCAFs, iCAFs, and immune-regulatory states—and their surrounding malignant cells, infiltrating leukocytes, and the ECM underscores the complexity of the tumor microenvironment (14, 15). Specifically, the TGF-β-driven myofibroblastic program is frequently associated with T-cell exclusion and resistance to immune checkpoint blockade (26). In contrast, emerging evidence suggests that certain immune-modulatory CAF states may exert context-dependent regulatory functions or even latent anti-tumor effects (27). Accordingly, the present review provides a systematic synthesis of existing literature on CAFs, with the objective of establishing a robust theoretical framework to facilitate precision oncology and targeted interventions in lung cancer.

## 2 Mechanism of action of CAFs

### 2.1 Mechanisms of CAFs-mediated extracellular matrix remodeling and mechanical signal transduction

Evidence from solid-tumor mechanobiology and pan-cancer stromal studies indicates that CAFs serve as ECM dyshomeostasis within the TME. By sustaining hyper-biosynthesis of collagen, Fibronectin (FN), proteoglycans, and Hyaluronic acid (HA), CAFs facilitate the formation of a pathognomonic dense desmoplastic stroma. Beyond passive matrix deposition, these cells actively remodel tumor mechanical boundaries through actomyosin-mediated traction forces, thereby modulating cancer cell mechanotransduction and morphology via physical compression (28). This reticular densification of the ECM promotes the accumulation of solid stress, which subsequently compromises microvascular integrity and intensifies intratumoral hypoxia, ultimately establishing a formidable barrier to therapeutic delivery (29). At the cellular state level, ECM deposition and contractile programs are preferentially associated with myCAF and ECM-producing CAF states rather than being uniformly distributed across all CAF populations. NSCLC-focused single-cell transcriptomic and spatial imaging studies have demonstrated that both MYH11^+^αSMA^+^ and FAP^+^αSMA^+^ CAF populations are associated with T-cell exclusion. The former are more prevalent in early lesions and localize along the periphery of cancer cell clusters, whereas the latter are enriched in advanced tumors, forming stromal plaques or multilayered stromal structures surrounding tumor nests. Although both populations are accompanied by dense, directionally aligned fiber deposition, they differ in matrix composition: MYH11^+^αSMA^+^ CAFs preferentially engage COL4A1/COL9A1 programs, whereas FAP^+^αSMA^+^ CAFs are biased toward COL11A1/COL12A1 programs (30). Hanley et al. further demonstrated, through single-cell analyses of NSCLC, that myofibroblast-like CAF populations are increased in both LUAD and LUSC compared with normal lung tissue, with a more pronounced expansion observed in LUSC. Moreover, elevated myofibroblast-like CAF abundance in LUAD was associated with poorer patient prognosis (31). Therefore, collagen crosslinking, FN alignment, and HA accumulation should not be regarded as universal features of all CAF populations. Rather, these stromal characteristics are more accurately interpreted as consequences of microenvironments dominated by myCAF or ECM-producing CAF states, including FAP^+^αSMA^+^, MYH11^+^αSMA^+^, and POSTN^+^ CAF subsets. Accumulating evidence shows that ECM abundance alone does not fully explain tumor invasiveness. ECM architecture and mechanical properties also play critical roles. These features include collagen crosslinking, fiber alignment, pore structure, and local stiffness. Together, they regulate tumor-cell migration, invasion, and therapeutic resistance. Experimental and clinical studies show that collagen crosslinking enhances integrin-dependent mechanotransduction. Aligned collagen or fibronectin fibers can promote directional invasion. Pore-scale matrix architecture can also influence cancer-cell escape and invasive phenotypes (32–34).

The lysyl oxidase family includes LOX and LOXL1 to LOXL4. This family forms a major enzymatic system that is a major mediator of collagen crosslinking and matrix stabilization. These copper-dependent amine oxidases catalyze oxidative deamination of lysine or hydroxylysine residues in collagen and elastin. This reaction promotes covalent crosslink formation, collagen fiber maturation, and ECM stiffening (32, 33, 35). Collectively, LOX family activity provides a biochemical link between ECM-producing CAF states, collagen crosslinking, and stromal stiffening. In lung cancer, LOX family members may link matrix remodeling to invasive behavior. E-box-binding homeobox 1 (ZEB1) induces LOXL2-mediated collagen stabilization and ECM deposition. This process promotes lung cancer invasion and metastasis (36). Tumor-derived LOXL2 can also activate stromal fibroblasts through integrin engagement and FAK signaling. This activation reinforces fibroblast activity and fibrotic stromal remodeling (37). Therefore, LOX family enzymes can be viewed as active mediators of the CAF-controlled mechanobiological niche.

At the invasive front, CAFs guide the directional alignment of FN and collagen fibers. This alignment helps tumor cells move more efficiently along matrix interfaces (38, 39). Moreover, CAFs exhibit a dualistic capacity for matrix regulation, they construct robust physical barriers while simultaneously deploying protease networks to liberate sequestered growth factors, thereby sustaining a non-equilibrium steady state characterized by both high density and structural plasticity (40–42). Recent single-cell and spatial transcriptomic studies of NSCLC have identified a POSTN^+^ myCAF-like subset. This subset is enriched in advanced NSCLC and is associated with immunosuppression and tumor progression. POSTN^+^ CAFs are also located near SPP1^+^ macrophages. This spatial relationship suggests that POSTN^+^ CAFs may connect ECM remodeling and mechanotransduction with myeloid-driven immune suppression (43).

**Figure 1:**
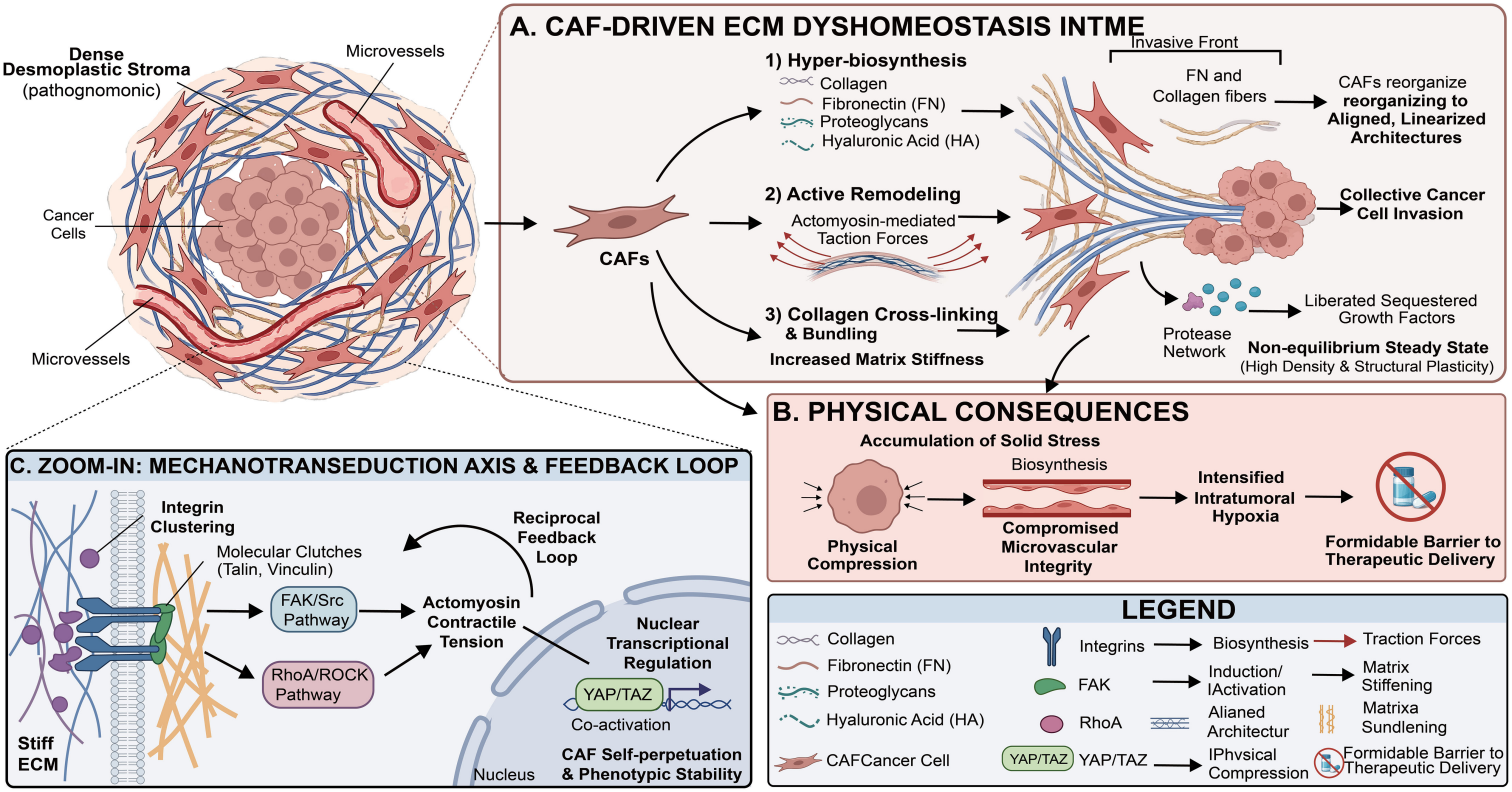
CAF-driven ECM modelingremodelling and mechanotransduction in lung cancer. **(A)** shows CAF-mediated ECM dyshomeostasis through increased collagen, fibronectin, proteoglycan, and hyaluronic acid biosynthesis, together with traction-force-dependent modelingremodelling. **(B)** shows physical consequences, including matrix stiffening, solid stress accumulation, vessel compression, hypoxia, and impaired therapy delivery. **(C)** shows integrin-mediated mechanotransduction through FAK/Src, RhoA/ROCK, and YAP/TAZ signaling, which maintains CAF activation and tumor resistance.

Mechanotransduction fundamentally centers on a coordinated signaling axis comprising integrins, Focal adhesions (FAs), cytoskeletal tension, and nuclear transcriptional regulation. Upon clustering, integrins employ molecular clutches, such as Talin and Vinculin, to transduce stiffness signals from the ECM to the actin cytoskeleton (44). This mechanical input is subsequently propagated and amplified through the Focal adhesion kinase (FAK)/Proto-oncogene tyrosine-protein kinase Src (Src) and Ras homolog family member A (RhoA)/Rho-associated protein kinase (ROCK) pathways, which intensify contractile tension and cultivate a reciprocal “matrix stiffening-tension enhancement” feedback loop (34, 45). As pivotal transcriptional co-activators, YAP and Transcriptional coactivator with PDZ-binding motif (TAZ) sense signals from the rigid interstitium to drive further matrix remodeling, ultimately reinforcing the self-perpetuation and long-term phenotypic stability of CAFs (46) (**Figure 1**). More recent reviews also support the role of ECM stiffness, FAK signaling, and YAP/TAZ activity in tumor progression, therapeutic resistance, and fibroblast-driven tissue remodeling (47, 49). Available evidence supports a close relationship between CAF-mediated ECM remodeling, matrix stiffness, and mechanotransduction. However, the strength of this support differs across experimental systems. *In vitro* matrix models, CAF-tumor cell co-culture systems and animal models have shown that ECM stiffness, integrin signaling, FAK/Src activation, RhoA/ROCK signaling and YAP/TAZ activity can promote matrix remodeling and tumor invasion (34, 47–49). Human NSCLC studies based on single-cell profiling, spatial imaging and multiplex tissue analysis further suggest an association between specific CAF states, aligned ECM programs and T-cell exclusion (30, 31). However, most patient-derived studies remain correlative and cannot fully establish causality. Thus, ECM stiffness and mechanotransduction may represent important and context-dependent contributors to CAF-driven tumor progression, while their direct therapeutic targeting in lung cancer remains translationally exploratory.

### 2.2 Regulatory role of CAF-derived paracrine signaling in lung cancer cell plasticity

CAFs orchestrate a continuous reconfiguration of the signaling topology within tumor cells by secreting an intricate secretome comprising cytokines, growth factors, and chemokines. This sustained ligand availability augments tumor cell proliferation, Epithelial-mesenchymal transition (EMT), invasion, and the maintenance of stemness, collectively facilitating the expansion of chemoresistant clones under therapeutic pressure. In lung cancer, NSCLC-focused functional studies and broader cancer biology evidence converge on intracellular nodes, including Signal transducer and activator of transcription 3 (STAT3), SMAD, Mitogen-activated protein kinase (MAPK)/Extracellular signal-regulated kinase (ERK), and Phosphoinositide 3-kinase (PI3K)/Protein kinase B (AKT) (50, 51). Such paracrine interactions are predominantly mediated via the Interleukin-6 (IL-6)–JAK/STAT3, TGF-β, Hepatocyte growth factor (HGF)–MET, and C-X-C motif chemokine ligand 12 (CXCL12)–C-X-C Motif Chemokine Receptor 4 (CXCR4) signaling axes (52–55).

#### 2.2.1 The IL-6/JAK/STAT3 signaling cascade

Within the IL-6/JAK/STAT3 signaling cascade, CAF-derived IL-6 engages the Interleukin-6 receptor (IL-6R)/gp130 receptor complex to activate JAK kinases, which subsequently induces the phosphorylation and nuclear translocation of STAT3. This nuclear accumulation upregulates the transcription of genes critical for cell survival, invasion, and the EMT, thereby augmenting the migratory and invasive capabilities of tumor cells while driving their mesenchymal phenotype (13). Functioning as a master inflammatory transcriptional hub, STAT3 integrates microenvironmental stressors into a stable state of cellular plasticity, thereby transitioning cancer cells into an anti-apoptotic, therapy-tolerant survival mode (56). Beyond its established roles in EMT and survival signaling, STAT3 activation also promotes tumor metabolic reprogramming. Constitutively active STAT3 can drive cancer cells toward aerobic glycolysis while reducing mitochondrial oxidative activity. This metabolic shift is partly mediated by hypoxia-inducible factor-1α (HIF-1α) induction and altered mitochondrial gene expression (57, 58). In cytokine-responsive cells, IL-6-induced STAT3 activation upregulates key glycolytic regulators, including hexokinase 2 (HK2) and 6-phosphofructo-2-kinase/fructose-2,6-bisphosphatase-3 (PFKFB3). These changes enhance glucose utilization and lactate production (59, 60). In NSCLC, inhibition of the IL-6/JAK/STAT3 pathway attenuates glycolytic activity in lung cancer cells. Reduced STAT3 signaling may downregulate glycolysis-associated regulators. This effect limits glucose utilization, lactate production, and the metabolic support required for rapid proliferation. Metabolic inhibition may also impair migration and invasion. Glycolytic adaptation provides energy and biosynthetic intermediates that support cytoskeletal remodeling, EMT-related plasticity, and survival under microenvironmental stress (61).

However, the strength of evidence varies across experimental contexts. Most direct evidence supporting CAF-derived IL-6 originates from conditioned-medium experiments, co-culture systems, and xenograft models. More specifically, CAF-conditioned medium studies and CAF–tumor cell co-culture studies have shown that IL-6/JAK/STAT3 activation promotes EMT in NSCLC cells. This activation also increases migration, metastatic potential, and chemoresistance (62, 63). *In vivo* evidence further supports the role of IL-6-related CAF secretory programs in NSCLC growth (64). Patient-derived evidence remains largely correlative. This evidence mainly comes from bulk transcriptomic signatures, immunohistochemistry, serum-based inflammatory indicators, and inferred inflammatory CAF states. These data support a link between IL-6 activity and an inflammatory tumor microenvironment. However, these data do not prove that CAFs are the exclusive source of IL-6 production in patient tumors. Therefore, IL-6 should not be considered a CAF-specific signal. Instead, IL-6 should be viewed as a central inflammatory mediator produced by multiple cellular compartments. CAFs represent an important stromal source of IL-6 in many inflammatory tumor microenvironments. In CAF-enriched niches, CAF-derived IL-6 may substantially promote JAK/STAT3 activation in tumor cells. This signaling promotes EMT, metabolic adaptation, and therapy-tolerant phenotypes. IL-6 can be produced by inflammatory CAFs, tumor cells, macrophages, endothelial cells, and other stromal populations. Consequently, increased IL-6 activity in patient samples does not necessarily indicate that CAFs are the dominant source. Moreover, IL-6 signaling frequently interacts with IL-1β, CXCL8, CXCL12, TGF-β, and myeloid suppressive pathways, which may attenuate the therapeutic efficacy of single-cytokine blockade.

#### 2.2.2 The TGF-β signaling axis

The TGF-β signaling axis represents a paramount paracrine pathway through which CAFs orchestrate EMT, heightened cellular plasticity, and therapeutic recalcitrance in lung cancer cells. Upon ligand-receptor engagement with Transforming growth factor-beta receptor 1/2 (TGFBR1/2), the canonical pathway triggers the nuclear translocation of the SMAD2/3–SMAD4 heteromeric complex. This transcriptional reprogramming upregulates master EMT transcription factors, including SNAIL and Zinc finger ZEB, which subsequently reorganize cellular polarity and intercellular junctions while driving the characteristic molecular shift from E-cadherin to Vimentin expression. Simultaneously, TGF-β signaling bifurcates into non-canonical branches by coupling with MAPK, PI3K/AKT, and Rho-GTPases to synergistically augment cytoskeletal remodeling and stress-responsive adaptation (65, 66). TGF-β-associated CAFs often exhibit increased expression of ECM- and myofibroblast-associated genes. These genes include ACTA2, TAGLN, MYH11, COL1A1, COL1A2, COL11A1 and POSTN. These CAF states promote tumor-cell invasion and plasticity through ECM deposition, contractile activity and SMAD-dependent transcriptional programs. Spatial transcriptomic and histological studies of human lung cancer show that CAF populations with active stromal programs are closely associated with T-cell exclusion. Recent studies have linked TGF-β-associated CAF programs with immune suppression and resistance to immune checkpoint blockade. Krishnamurty et al. identified LRRC15 as a TGF-β-dependent marker of myofibroblasts.

Their study demonstrated that genetic ablation of LRRC15-positive CAFs reduced fibroblast burden while restoring CD8^+^ T-cell effector function and enhancing responses to anti-PD-L1 therapy in preclinical models (67). In lung cancer, Qi et al. identified LRRC15-positive CAFs as a tumor-enriched CAF subset linked to poor prognosis. Their study showed that loss of LRRC15 in CAFs reduced ECM production. It also inhibited CD206-positive macrophage polarization, enhanced CD8-positive T-cell cytotoxicity, and suppressed lung cancer progression in mouse models (68). The same study developed an LRRC15-targeted TGF-β trap. This treatment delayed tumor growth in a KPS lung cancer model. These findings provide direct functional evidence that LRRC15-positive TGF-β-associated CAFs can link ECM remodeling to myeloid-mediated immunosuppression.

Additional spatial analyses have further supported the clinical relevance of this axis. Lee and Lee identified fibroblast TGF-β-related spatial tumor ecotypes in LUAD. They also validated the association between these ecotypes and ICB response across four independent LUAD immunotherapy cohorts. The TGF-β-high fibroblast ecotype showed higher immune-exclusion scores. This ecotype was associated with shorter progression-free survival following ICB. These findings support stromal TGF-β activity as a potential biomarker for immune-excluded NSCLC (69).

Thus, TGF-β-associated CAF states represent a critical mechanistic axis connecting EMT, matrix remodeling, immune exclusion and therapeutic resistance. However, their functional effects remain shaped by tumor type, spatial niche and experimental context.

Compared with IL-6, the TGF-β axis is supported by stronger spatial and functional evidence, particularly because TGF-β-enriched stromal programs have been linked to impaired T-cell penetration into tumor nests and reduced responsiveness to PD-L1 blockade (21). Nevertheless, the interpretation of TGF-β signaling requires caution. TGF-β exerts stage-dependent and cell-type-specific effects, and its biological functions may differ among tumor cells, fibroblasts, immune cells, and endothelial cells (54, 61). During early tumorigenesis or tissue repair, TGF-β may contribute to growth control, immune regulation, and tissue homeostasis. In established tumors, however, it may promote extracellular matrix remodeling, immune suppression, and metastatic competence. This context dependence may partly explain why broad TGF-β inhibition does not consistently translate into clinical benefit. In addition, PD-L1 expression alone may be insufficient to identify tumors in which TGF-β-driven CAF programs represent the dominant mechanism of immune exclusion.

#### 2.2.3 The HGF/MET axis

CAFs represent the predominant reservoir of HGF within the TME (70). The engagement of HGF with the mesenchymal-epithelial transition factor (c-MET) triggers receptor dimerization and autophosphorylation, which subsequently activates downstream signaling cascades, including the RAS/MAPK and PI3K/AKT pathways, to drive tumor cell proliferation, survival, and stress adaptation. Consequently, even if upstream Receptor tyrosine kinases (RTKs) such as Epidermal growth factor receptor (EGFR) are effectively suppressed by Tyrosine kinase inhibitors (TKIs) or monoclonal antibodies, MET can circumvent this inhibition by re-initiating essential downstream signals. This bypass activation serves as a pivotal non-cell-autonomous component of resistance against EGFR-targeted therapies (71). However, the evidence base remains uneven. Most direct mechanistic evidence for the CAF-related HGF/MET axis comes from lung fibroblast or CAF-conditioned medium experiments. This evidence also comes from fibroblast-tumor cell interaction models, co-culture systems, xenografts, PDX-based analyses, and selected EGFR-TKI-resistant models (70–72). In contrast, patient-level evidence is more limited and is often confounded by tumor-intrinsic resistance mechanisms, including MET amplification, MET exon 14 skipping, HER family activation, AXL activation, and histologic transformation (70, 71). Current patient-derived support mainly comes from functional analyses of patient-derived lung CAFs. This support also comes from single-cell profiling and PDX-based resistance studies. Prospective biomarker-defined clinical trials have not yet provided the main evidence. Recent single-cell and PDX-based studies have identified specific CAF subsets, such as CTHRC1-positive CAFs, that contribute to EGFR-TKI resistance through metabolic reprogramming and TGF-β/SMAD-related signaling. These findings support the existence of resistance-associated CAF states, but they also indicate that HGF/MET signaling represents only one component of a broader stromal resistance network. Therefore, HGF/MET signaling should be presented as a context-dependent paracrine resistance mechanism rather than as a universal CAF-specific therapeutic target in NSCLC (72).

#### 2.2.4 The CXCL12/CXCR4 axis

The CXCL12/CXCR4 axis exerts pleiotropic effects within lung cancer CAFs, encompassing the promotion of cell migration, the establishment of immune exclusion, and the facilitation of CAF expansion (73–75). Regarding tumor cell motility, CXCL12/CXCR4 signaling activates downstream PI3K/AKT, MAPK/ERK, and FAK/Src pathways, thereby promoting cytoskeletal remodeling, focal adhesion dynamics, migration, and invasion in lung cancer cells (76, 77). From an immunological perspective, CAF-secreted CXCL12 orchestrates a spatially compartmentalized immune landscape by recruiting suppressive myeloid cells and sequestering effector immune cells within the stroma. This process, coupled with the induction of M2-like macrophages polarization, culminates in a functional immune exclusion zone that hinders the penetration of anti-tumor cells into the cancer nests (73, 78). Furthermore, CXCL12 serves as a self-amplifying stimulus that drives the phenotypic reprogramming of normal fibroblasts into CAF-like cells. This maintenance of the CAF lineage is intrinsically linked to STAT3 signaling, suggesting that the CXCL12/STAT3 axis concurrently sustains pro-invasive signaling and preserves the CAF population while operating in tandem with Programmed death-ligand 1 (PD-L1)-mediated immune evasion (73, 79).

**Figure 2:**
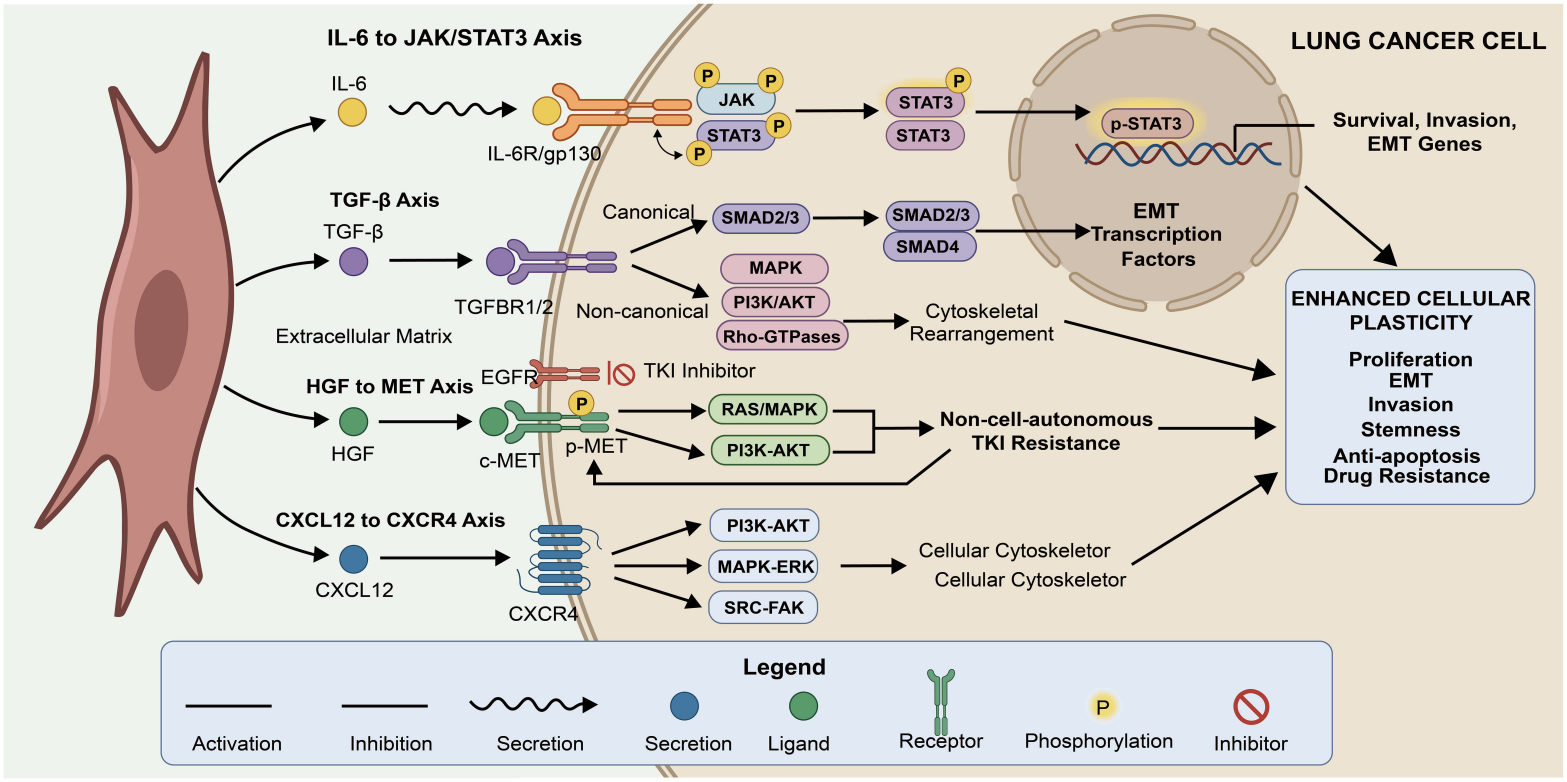
CAF-derived paracrine signaling regulates lung cancer cell plasticity and therapeutic resistance. CAF-secreted IL-6, TGF-β, HGF, and CXCL12 engage IL-6R/gp130, TGFBR1/2, MET, and CXCR4 on lung cancer cells. These axes activate JAK/STAT3, SMAD2/3, MAPK/ERK, PI3K/AKT, and SRC/FAK modules. The outputs include STAT3-driven survival, TGF-β-mediated EMT, HGF/MET-dependent bypass signaling, and CXCL12/CXCR4-associated migration, collectively promoting invasion, stemness, anti-apoptosis, and resistance to EGFR-targeted therapy.

The pro-migratory and macrophage-related functions of the CXCL12/CXCR4 axis are supported mainly by fibroblast or CAF co-culture systems, chemotaxis assays, macrophage polarization assays, pathway inhibition experiments, xenograft models, and genetic mouse models (73–75, 78). Patient-derived support mainly comes from NSCLC tissue analyses. These analyses show associations among CD248-positive CAFs, CXCL12 expression, M2-like macrophage infiltration, and tumor progression (74, 75). The principal limitation of this axis is the substantial redundancy of chemokine signaling. The CXCL12/CXCR4 axis may interact with CXCL8/CXCR2, CCL2/CCR2, and other myeloid recruitment pathways. Consequently, blockade of a single chemokine axis may be insufficient to restore durable antitumor immunity. In addition, the available evidence in NSCLC remains dominated by preclinical models and patient tissue-based associations, whereas clinical evidence is largely extrapolated from early-phase trials in other solid tumors or from combination strategies designed to overcome immune exclusion. These limitations suggest that CXCL12/CXCR4 should be evaluated preferentially in spatially defined immune-excluded tumors (**Figure 2**).

### 2.3 CAF-mediated immunosuppression and T-cell exclusion mechanisms

A defining feature of the lung cancer TME is the sequestration of T cells within the peritumoral stroma, which precludes their infiltration into the malignant parenchyma. Current evidence identifies CAFs as the central stromal architects of this T-cell exclusion phenotype. This regulatory influence is primarily exerted through two distinct yet complementary mechanisms. First, CAFs modulate the local immune landscape by secreting a repertoire of chemokines, inflammatory mediators, and immunomodulatory factors that favor the recruitment and expansion of suppressive myeloid cell populations. Concurrently, CAFs sustain persistent ECM deposition and drive aberrant tissue mechanics, thereby establishing densified spatial barriers that physically hamper T-cell motility (79–81). Collectively, these biochemical and biophysical impediments attenuate the sensitivity of lung cancer to Immune checkpoint blockade (ICB) therapies. This interpretation is supported by two complementary levels of evidence. Patient-derived single-cell transcriptomic, spatial transcriptomic, and multiplex imaging studies define the spatial association between CAF-rich stromal niches and T-cell exclusion in human NSCLC tissues (30, 82). Functional studies using co-culture systems, xenograft models, and genetically modified mouse models further support the causal capacity of specific CAF programs to reshape immune infiltration (83–85). Therefore, CAF-mediated immune exclusion should be viewed as a clinically relevant mechanism with variable degrees of causal validation across experimental systems.

Single-cell transcriptomics, spatial transcriptomics and high-dimensional tissue imaging have provided mechanistic insight into CAF-mediated immune regulation in lung cancer. Using multiplexed spatial profiling, Pellinen et al. showed that CAF composition in NSCLC is closely associated with oncogenic driver status, PD-L1 expression, CD163^+^ cell density and patient outcome (82). Grout et al. further identified a CAF population in human lung cancer linked to T-cell exclusion, characterized by matrix-remodeling programs and distinct spatial positioning. By connecting CAF transcriptional states, ECM architecture and the peripheral localization of T cells, this study supports a model in which CAF-driven spatial matrix remodeling restricts T-cell infiltration into the tumor parenchyma (30). These patient-derived studies provide strong spatial evidence because they analyze intact human tumor tissues (30, 82). However, they remain limited in causal inference. CAF accumulation in immune-excluded regions may reflect active stromal regulation, but it may also coexist with tumor-intrinsic immune evasion, hypoxia, myeloid inflammation, or previous treatment exposure (30, 82, 86).

The orchestration of suppressive myeloid cell networks constitutes a cornerstone of CAF-mediated immunomodulation in lung cancer. By secreting C-C motif chemokine ligand 2 (CCL2), CAFs facilitate the recruitment of C-C motif chemokine receptor 2 (CCR2) monocytes and subsequently drive their differentiation into Monocytic myeloid-derived suppressor (M-MDSCs). These myeloid populations impair antitumoral immunity by generating Reactive oxygen species (ROS) that suppress CD8^+^ T-cell proliferation and Interferon-gamma (IFN-γ) secretion (87, 88). Furthermore, the CAF-driven accumulation of MDSCs and immunosuppressive Tumor-associated macrophages (TAMs) not only hampers dendritic cell maturation and antigen presentation efficiency but also persistently blunts CD8^+^ T-cell cytotoxicity through oxidative stress, L-arginine depletion, and the release of immunosuppressive cytokines (83, 86). Beyond indirect myeloid remodeling, specific CAF subpopulations exert direct control over the local immune landscape. CD248 is mainly expressed in CAFs from NSCLC tissues. CD248^+^ CAFs secrete CXCL12 and promote macrophage recruitment through CXCR4. They also drive macrophages toward an M2-like state. This shift is marked by increased expression of CD163, CD206 and IL-10. Blocking CXCR4 strongly reduces the chemotactic activity of M2-like macrophages (75). Further studies show that M2-like macrophages induced by CD248^+^ CAFs secrete TGF-β and promote EMT, migration and invasion in NSCLC cells. This process is accompanied by downregulation of E-cadherin and upregulation of N-cadherin, Vimentin, p-Smad2/3 and Twist-related protein 1 (TWIST1) (77). In addition, CD248 upregulates PD-L1 expression in CAFs through the FAK/Src/JNK/c-Jun axis, thereby suppressing CD8^+^ T-cell function and promoting immune evasion in NSCLC (78). These findings broaden the CCL2/CCR2-mediated MDSC model by showing that CAF-mediated immunosuppression is not confined to a single chemokine pathway. Distinct CAF states may engage different ligand-receptor axes to recruit macrophages, induce immune checkpoint expression, and reinforce local immune suppression (75, 78). However, the CD248-centered circuit remains supported mainly by preclinical and tissue-association evidence. Its value as a predictive biomarker for ICB response requires validation in larger, treatment-annotated NSCLC cohorts (78, 82).

**Figure 3:**
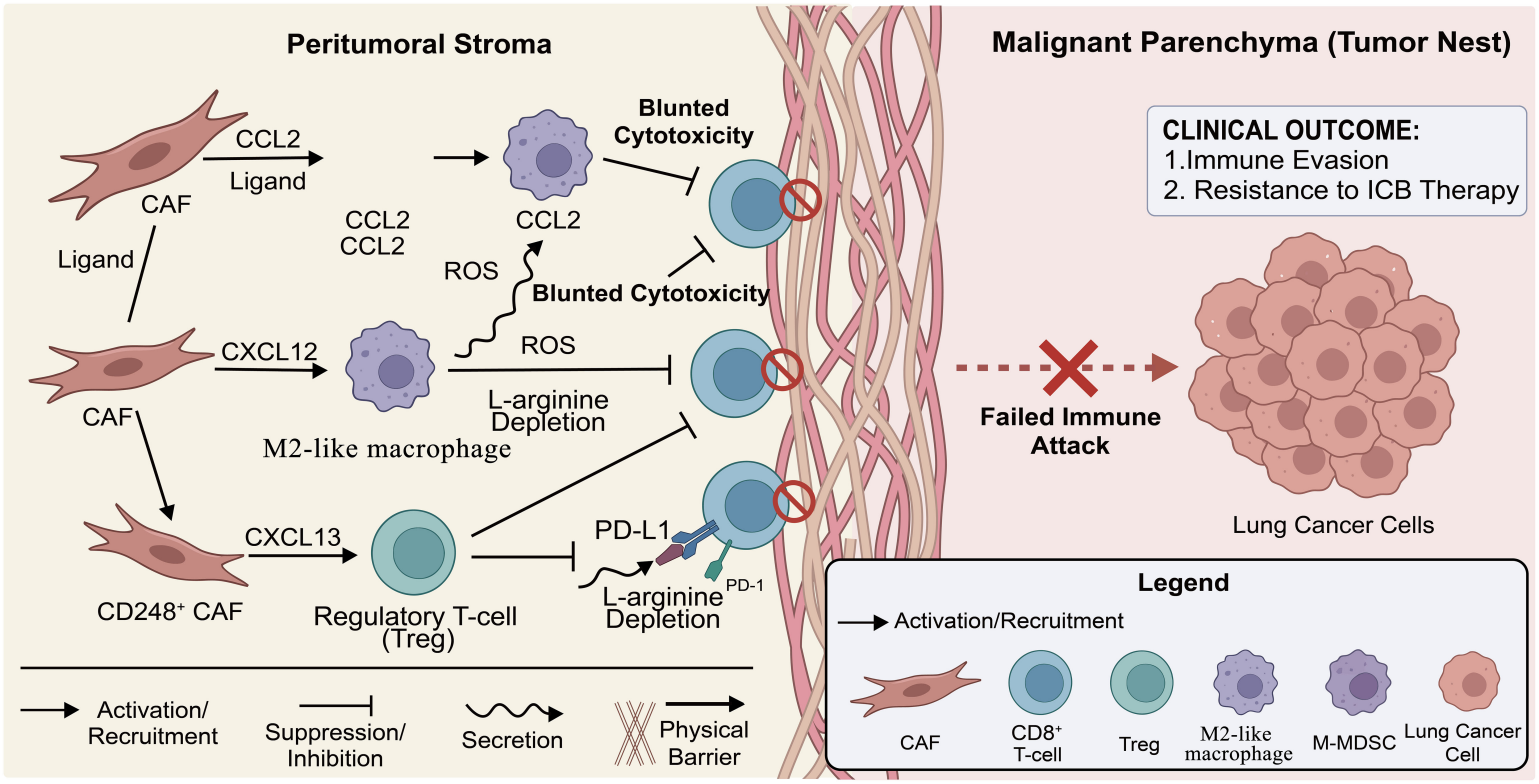
CAF-mediated T cell exclusion and immunosuppression in lung cancer. The left region depicts peritumoral CAFs and CD248^+^ CAFs secreting CCL2, CXCL12, and CXCL13. These signals recruit M-MDSCs, polarize M2-like macrophages, expand Tregs, and increase PD-L1 related suppression. The central collagen barrier limits CD8^+^ T cell entry into the right tumor nest, resulting in failed immune attack and resistance to ICB therapy.

Single-cell and spatial transcriptomics have also clarified how CAFs interact with myeloid cells to promote immunosuppression. Chen et al. identified a POSTN^+^ myCAF subset in NSCLC. This subset was enriched in advanced tumors and showed expression features linked to ECM remodeling, tumor invasion and immune suppression (43). Spatial transcriptomics and multiplex immunohistochemistry showed that POSTN^+^ CAFs were located near SPP1^+^ macrophages. These spatial patterns were associated with reduced T-cell infiltration and an exhausted T-cell phenotype (43). Xiao et al. further integrated bulk RNA-seq, single-cell RNA-seq, spatial transcriptomics, multiplex immunofluorescence and conditional knockout mouse models. Their study showed that FAP^+^ fibroblasts and SPP1^+^ macrophages interact strongly in NSCLC tissues. The VCAN-ITGB1 axis may mediate this interaction. Spatial analysis placed these interaction niches around the tumor core. These niches were associated with the accumulation of CD4^+^ T cells, CD8^+^ T cells and NK cells at the tumor margin. Macrophage-specific Spp1 deletion increased CD3^+^ and CD8^+^ T-cell infiltration and reduced tumor growth (84). This integrated evidence provides stronger support than spatial association alone because it combines patient tissue mapping with functional perturbation. However, the causal mechanism is best interpreted as a multicellular CAF-macrophage interaction. The available data do not support a purely CAF-autonomous model of immune exclusion (43, 84). Additionally, a reciprocal functional coupling exists between CAFs and T cells within the lung cancer microenvironment. Under TGF-β-dependent conditions, activated T cells can induce CAFs to enhance C-X-C motif chemokine ligand 13 (CXCL13) expression, a process associated with Regulatory T-cell (Treg) expansion and restricted effector T-cell activation (89). Finally, the profound densification and altered topological orientation of collagen fibers create biophysical impediments that restrict T-cell infiltration, resulting in their persistent sequestration within the peritumoral stroma (90) (**Figure 3**).

Notably, the immunoregulatory role of apCAFs in lung cancer is highly context dependent. apCAFs are defined by elevated expression of MHC-II and antigen-presentation-associated molecules. In lung cancer, MHC-II^+^FAP^+^CD45^−^ fibroblasts can form local immune niches enriched with CD4^+^ T cells within tumor tissues and directly activate effector CD4^+^ T cells through MHC-II-dependent antigen presentation. Mechanistically, lung cancer apCAFs not only mediate local antigen presentation but also produce C1q, which supports the survival of tumor-infiltrating CD4^+^ T-cells through the C1q–C1qbp axis. Fibroblast-specific loss of MHC-II weakens local CD4^+^ T-cell immunity and accelerates lung cancer progression, indicating that apCAFs can sustain antitumor immunity in specific contexts (90). Recent pan-cancer spatial analyses further divide apCAFs into mesothelial-like apCAFs located near cancer cells and fibrocyte-like apCAFs positioned close to lymphocyte-enriched regions. Both states can upregulate SPP1 and contribute to tumor initiation, metastasis and therapeutic resistance (91). Therefore, apCAFs in the lung cancer immune microenvironment are best described as CAF states with local antigen-presenting and immune-regulatory functions. Their effects depend on cellular origin, spatial location, antigen-presenting capacity and the immune cells in their neighborhood.

Overall, the evidence for CAF-mediated immunosuppression in lung cancer is strongest when single-cell profiling, spatial localization, multiplex imaging and functional perturbation are combined. The main limitation is that many studies remain observational and cannot fully distinguish CAF-driven causality from immune-cell-driven or tumor-cell-driven mechanisms. Another limitation is CAF depletion or broad CAF inhibition may eliminate both immunosuppressive and immune-supportive CAF states.

### 2.4 Mechanisms of CAF-sEV and exosome-mediated bidirectional communication in lung cancer

**Figure 4:**
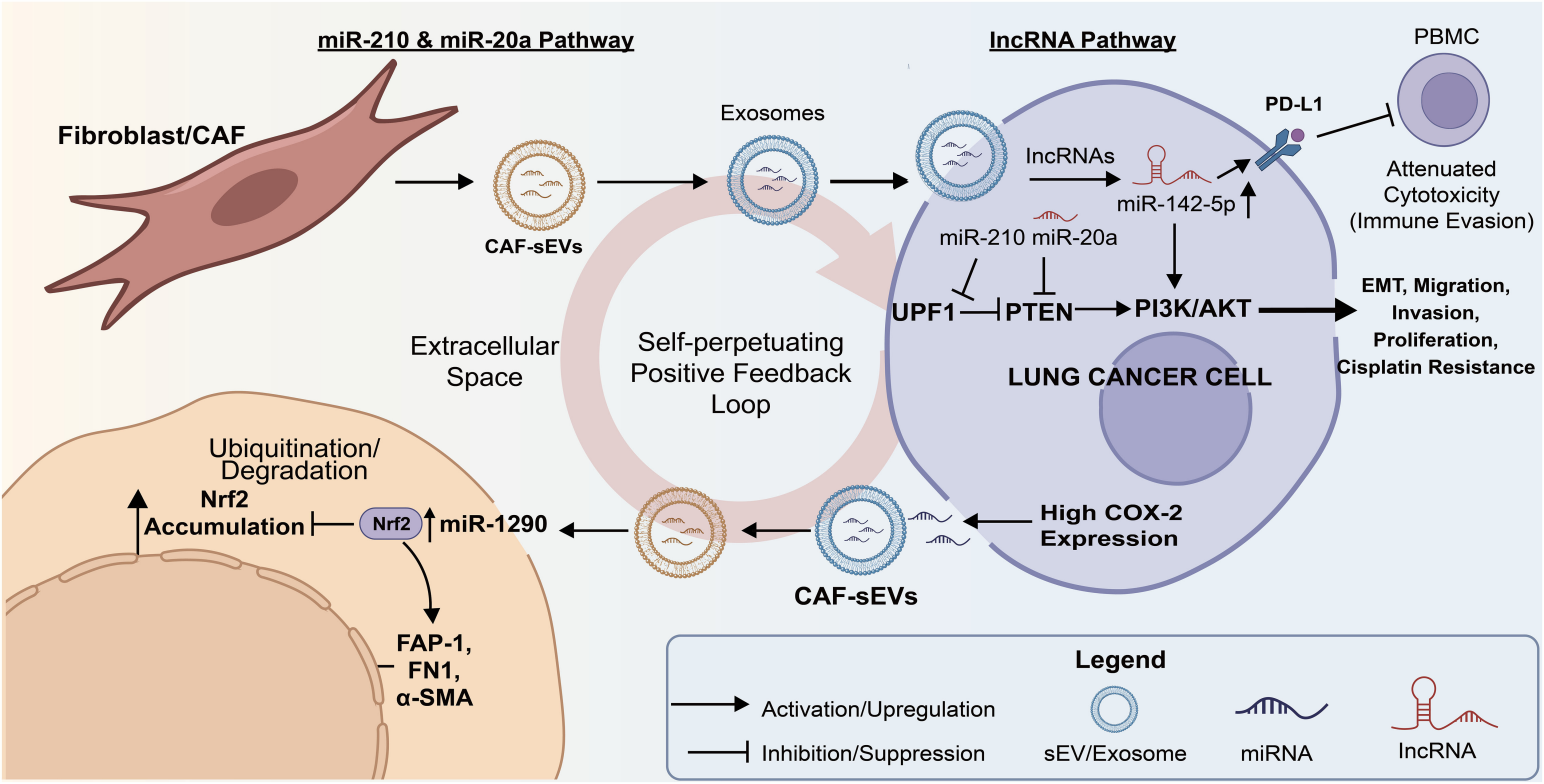
sEV-mediated bidirectional communication between CAFs and lung cancer cells. The upper route shows CAF-derived sEVs transferring miR-210, miR-20a, and lncRNAs to suppress UPF1 or PTEN, activate PI3K/AKT signaling, and increase PD-L1 expression. The lower route shows tumor sEVs carrying miR-1290 to fibroblasts, causing Nrf2 accumulation and FAP-1, FN1, and α-SMA upregulation. Together, these routes reinforce tumor plasticity, immune evasion, drug resistance, and a pro-tumorigenic feedback loop.

Cancer-associated fibroblast (CAF)-derived Small extracellular vesicles (sEVs) represent pivotal paracrine vehicles that drive phenotypic remodeling within the lung cancer microenvironment. Evidence suggests that these vesicles are efficiently internalized by lung cancer cell lines, such as A549 and H1299, to deliver functional payloads including microRNAs (miRNAs), Long non-coding RNAs (lncRNAs), and proteins. This cargo-mediated delivery subsequently reconfigures the invasive, metabolic, and therapeutic response programs of the recipient cells. For instance, CAF-derived exosomes enriched with miR-210 target Up-frameshift suppressor 1 (UPF1) upon entering NSCLC cells, which inhibits Phosphatase and tensin homolog (PTEN) and activates the PI3K/AKT signaling axis to promote EMT, migration, and invasion (92). Similarly, exosomal miR-20a from CAFs downregulates PTEN to activate the PI3K/AKT pathway, thereby enhancing the proliferative capacity and cisplatin resistance of NSCLC cells (77). In the context of immune evasion, CAF-derived exosomal lncRNAs function as molecular decoys by sequestering miR-142-5p, which leads to the upregulation of PD-L1 expression in lung cancer cells. This mechanism effectively attenuates Peripheral blood mononuclear cell (PBMC)-mediated cytotoxicity, establishing a definitive link between stroma-derived exosomes and pulmonary immunosuppression (93). These studies provide direct cellular and molecular evidence. However, their conclusions are mainly based on selected vesicle cargoes and defined experimental systems. It remains uncertain whether these cargoes are consistently enriched in CAF-derived sEVs across heterogeneous NSCLC tissues, disease stages, and treatment backgrounds. Complementing the regulatory influence of CAFs, lung cancer cells also utilize exosomes to reciprocally modulate fibroblasts, ensuring their stable conversion into a CAF phenotype. Specifically, lung adenocarcinoma cells with high Cyclooxygenase-2 (COX-2) expression release exosomes laden with miR-1290. Upon entering fibroblasts, these vesicles inhibit the ubiquitination and degradation of nuclear factor erythroid 2-related factor 2 (Nrf2), leading to the upregulation of CAF activation and matrix remodeling markers, including Fibroblast activation protein 1 (FAP-1), Fibronectin 1 (FN1), and Alpha-smooth muscle actin (α-SMA) (94). Ultimately, this sEV-mediated bidirectional communication establishes a self-perpetuating positive feedback loop that progressively reinforces the pro-tumorigenic niche and drives disease progression (**Figure 4**). This bidirectional model is biologically plausible and is supported by experimental studies using tumor-cell-derived and CAF-derived vesicles. However, patient-derived evidence remains largely associative. Current analyses of tissue or circulating EVs often cannot reliably define the cellular origin of detected cargo. Such cargo may derive from CAFs, tumor cells, immune cells, endothelial cells, or other stromal compartments.

At present, evidence supporting the CAF-sEV axis remains less robust than evidence for ECM remodeling or TGF-β-driven immune exclusion. Most studies rely on isolated CAFs, EV isolation from conditioned medium, candidate cargo validation, and *in vitro* gain- or loss-of-function experiments. In addition, EV isolation methods, purity controls, CAF-source specificity, and cargo quantification remain poorly standardized across studies. Patient-level evidence is largely associative, and the CAF origin of circulating or tissue EV cargo is still difficult to confirm. Importantly, no CAF-sEV-targeted investigational new drug or Phase I clinical program has been reported in lung cancer. Therefore, the CAF-sEV axis should be viewed as a promising mechanistic and biomarker-oriented research direction, but its clinical translation remains at an early stage as summarized in **Table 1**.

**Table 1:** Integrated CAF subsets, signaling crosstalk, and therapeutic implications in lung cancer.

CAF subsets/states	Integrated signaling crosstalk	Major biological functions	Evidence level	Therapeutic implications
myCAFs/ECM-producing CAFs (αSMA^+^, FAP^+^αSMA^+^, MYH11^+^αSMA^+^, POSTN^+^, LRRC15^+^)	TGF-β–SMAD cooperates with integrin–FAK/Src, RhoA/ROCK and YAP/TAZ; non-canonical TGF-β converges with MAPK/ERK and PI3K/AKT.	ECM deposition, collagen/FN alignment, matrix stiffening, high interstitial pressure, invasion, drug-delivery barrier and T-cell exclusion.	Strong: supported by scRNA-seq, spatial profiling, multiplex imaging and mechanistic models.	Prioritize stromal normalization over broad depletion; consider matrix/mechanotransduction modulation and TGF-β-based ICB combinations in biomarker-defined tumors.
Inflammatory CAFs (iCAFs) (IL-6^+^, IL-1-responsive, CXCL8/CXCL12-high states)	IL-1β–IL-6/JAK/STAT3, CXCL8–CXCR2 and CXCL12–CXCR4 interact with TGF-β and myeloid-suppressive circuits.	Tumor-cell plasticity, EMT, survival signaling, therapy tolerance and myeloid recruitment.	Moderate: mainly co-culture, conditioned-medium, xenograft and correlative patient-tissue evidence.	Avoid single-cytokine blockade alone; combine chemokine/inflammatory targeting with ICB and stratify by iCAF activity, CRP/IL-6 status and immune-exclusion patterns.
Resistance-associated CAFs (HGF-high, CTHRC1^+^ CAFs)	CAF-derived HGF activates MET and downstream RAS/MAPK and PI3K/AKT, bypassing EGFR inhibition; overlaps with TGF-β/SMAD and metabolic resistance programs.	Tumor survival, stress adaptation, EMT-like plasticity and non-cell-autonomous resistance to EGFR-targeted therapy.	Moderate: strong preclinical support, but patient interpretation is confounded by tumor-intrinsic MET, AXL and histologic resistance mechanisms.	Use HGF–MET targeting selectively in stromal resistance contexts; pair with EGFR-TKIs only when biomarkers distinguish CAF-driven bypass from tumor-intrinsic MET alterations.
Immunoregulatory CAF (CXCL12^+^/CD248^+^ CAFs; FAP+ CAF–SPP1^+^ macrophage niches)	CXCL12–CXCR4 links PI3K/AKT, MAPK/ERK, FAK/Src and STAT3; CD248 and FAP^+^ niches couple CAFs with M2-like macrophages and SPP1 signaling.	M2-like macrophages recruitment, myeloid suppression, PD-L1 induction, impaired CD8^+^ T-cell function, exhausted T-cell phenotypes and immune exclusion.	Moderate to strong: supported by spatial multi-omics, tissue imaging, chemotaxis/blockade assays and functional mouse models.	Target spatially defined stromal-myeloid niches; combine CXCR4/CXCL12, SPP1- related strategies with PD-1/PD-L1 blockade.
Antigen-presenting CAFs (apCAFs) (MHC-II^+^, FAP^+^CD45^−^, C1q-producing states)	MHC-II-dependent antigen presentation cooperates with C1q–C1qbp signaling; context-specific apCAF-like states may also intersect with SPP1-rich niches.	Context-dependent immune regulation; some lung cancer apCAFs support local CD4^+^ T-cell survival and antitumor immunity.	Moderate: supported by human tissue studies, spatial analyses and functional perturbation of fibroblast MHC-II.	Preserve immune-supportive apCAF niches while selectively suppressing pro-tumor CAF programs.
CAF-sEV/exosome-producing CAF states	sEV cargo, including miR-210, miR-20a and lncRNA-related axes, activates PTEN–PI3K/AKT and PD-L1 pathways; tumor exosomes reinforce CAF activation via miR-1290–Nrf2.	EMT, migration, invasion, cisplatin resistance, immune escape, fibroblast activation and self-reinforcing CAF–tumor feedback.	Emerging: mostly EV isolation, cargo validation and *in vitro* gain/loss-of-function studies; CAF-source specificity remains limited.	Promising biomarker and theranostic direction; potential targets include sEV release, uptake and pathogenic cargo, but lung cancer clinical translation remains early.

This table summarizes major CAF subsets/states in lung cancer, emphasizing their representative signaling crosstalk, biological functions, evidence strength, and potential therapeutic implications. Evidence levels are interpreted according to the consistency of findings from patient-based multi-omics studies, spatial analyses, and experimental validation. CAF, cancer-associated fibroblast; myCAF, myofibroblastic CAF; iCAF, inflammatory CAF; apCAF, antigen-presenting CAF; ECM, extracellular matrix; NSCLC, non-small cell lung cancer; EMT, epithelial–mesenchymal transition; ICB, immune checkpoint blockade; sEV, small extracellular vesicle.

## 3 Potential therapeutic strategies targeting CAF-mediated pathogenesis in lung cancer

Emerging evidence underscores that CAFs represent a highly plastic cellular consortium rather than a singular, static therapeutic target. This population persistently orchestrates tumor progression and immune evasion through the assembly of biophysical ECM barriers, secretome-mediated cellular reprogramming, and the bidirectional regulatory dynamics of the CAF-sEV axis. Consequently, therapeutic paradigms targeting CAFs are shifting from non-selective cellular ablation toward the nuanced modulation of their functional states. It is critical to recognize that indiscriminate fibroblast depletion can paradoxically induce tumor dedifferentiation and exacerbate immunosuppressive signaling in certain models, thereby compromising overall survival benefits (95, 96). In contrast, strategies centered on the reversal of pathogenic phenotypes and stromal reprogramming demonstrate superior biological rationale and hold greater promise for clinical translation.

### 3.1 Intervention strategies targeting ECM physical barriers and mechanotransduction

To counteract the pathogenic influence of the ECM and the mechanical microenvironment, current therapeutic strategies aim to attenuate elevated IFP and reduce diffusion resistance. By enhancing tissue perfusion, these approaches facilitate the effective parenchymal infiltration of therapeutic agents and effector immune cells ^[22]^. The accumulation of HA represents a central component of this pharmacoperfusion barrier; thus, alleviating the mechanical burden through enzymatic HA degradation is considered a promising pathway for sensitizing tumors to chemotherapy and immunotherapy (97). For instance, exploratory clinical investigations into PEGPH20 combined with Programmed cell death protein 1 (PD-1) inhibitors for advanced NSCLC characterized by high HA expression underscore the preliminary feasibility of biomarker-stratified combinatorial regimens (98). Furthermore, the repurposing of established anti-fibrotic agents has demonstrated significant clinical potential. Pirfenidone, for instance, suppresses fibrotic transcriptional programs and modulates the reciprocal interaction between CAFs and the ECM, thereby augmenting anti-tumor efficacy (99, 100). nintedanib, a multi-target tyrosine kinase inhibitor, effectively inhibits fibroblast activation and pro-fibrotic signaling while simultaneously exerting anti-angiogenic effects. These dual actions optimize the biophysical state of the microenvironment, providing a robust theoretical basis for its synergy with immunotherapeutic or chemotherapeutic regimens (101, 102). The fundamental objective of these strategies is to optimize the physical functionality of the TME. By reducing intratumoral tension and improving drug accessibility, these interventions align with the clinical challenges of lung cancer, which is frequently characterized by pronounced spatial heterogeneity and restricted tissue penetration. Nevertheless, clinical evidence for HA degradation in combination with immunotherapy remains largely confined to exploratory studies of PEGPH20 plus pembrolizumab, which currently lack the consistency required to support universal clinical utility (103). Moreover, given the inherent heterogeneity of CAFs and the ECM, non-selective interventions may inadvertently dismantle the matrix-mediated physical constraints on tumor cells, potentially fostering an immunosuppressive milieu or accelerating disease progression (95, 96). Consequently, future research should prioritize the selective reprogramming of specific CAF subpopulations and matrix features while rigorously evaluating the real-world efficacy and safety profiles of agents such as pirfenidone and nintedanib in the lung cancer context.

### 3.2 Intervention strategies targeting paracrine signaling networks and immune microenvironment remodeling

#### 3.2.1 Intervention in the inflammatory cytokine network

For secretome-driven tumor-cell reprogramming, therapeutic strategies should target key inflammatory factors and chemokine pathways, thereby reducing immune exclusion and limiting the emergence of drug-tolerant cellular states. In the inflammatory cytokine network, researchers have paid close attention to IL-1β and its downstream IL-6/CRP pathway. In the CANTOS trial, canakinumab neutralized IL-1β and reduced systemic inflammation. The exploratory cancer analysis also linked canakinumab to lower lung cancer incidence and lower lung cancer mortality (104). Importantly, the CANTOS trial did not include patients with established advanced NSCLC. Therefore, its findings primarily reflect changes in lung cancer risk under chronic inflammatory conditions and do not directly demonstrate therapeutic benefit in advanced disease. Later CANOPY trials also did not confirm the clinical value of canakinumab in phase III treatment settings. In CANOPY-1, researchers added canakinumab to first-line pembrolizumab plus platinum-based doublet chemotherapy. This combination did not significantly extend progression-free survival or overall survival in patients with advanced NSCLC (105). CANOPY-2 further showed that canakinumab combined with docetaxel provided no additional survival benefit over docetaxel alone in patients with advanced NSCLC who had progressed after platinum-based doublet chemotherapy and immunotherapy (106).

These findings suggest that the limited efficacy of IL-1β blockade does not undermine the therapeutic relevance of inflammatory CAFs or iCAFs. Rather, it highlights the complexity and redundancy of pro-tumor inflammatory networks in advanced NSCLC. Several mechanisms may account for this outcome. First, IL-1β represents only one upstream node within the inflammatory cascade, whereas the advanced NSCLC microenvironment can sustain immunosuppression through IL-6, CXCL8, CXCL12, TGF-β and myeloid-derived suppressor cell-associated pathways. Second, the CANOPY trials mainly stratified patients according to clinical stage and treatment line, without incorporating CAF subtype states, IL-1β/IL-6/CRP inflammatory activity, high MDSC infiltration or spatial immune-exclusion phenotypes. Third, inflammatory signals in patient tumors may originate from multiple cellular compartments, including CAFs, tumor cells, myeloid cells and endothelial cells. Isolated IL-1β blockade may therefore be insufficient to remodel the functional state of the tumor microenvironment. Future strategies targeting inflammatory CAFs should avoid broad blockade of a single cytokine and instead integrate iCAF markers, systemic inflammatory indices, the extent of myeloid infiltration and spatial immune-localization features to identify patients whose tumors depend on IL-1β-associated inflammatory programs.

#### 3.2.2 Modulation of immune infiltration via chemokine axis blockade

The targeted inhibition of chemokine signaling axes is fundamental to the modulation of the intratumoral immune landscape. Specifically, the combination of the C-X-C Motif Chemokine Receptor 2 (CXCR2) antagonist navarixin and pembrolizumab has been shown to mitigate escape mechanisms driven by immunosuppressive cells, thereby facilitating CD8^+^ T-cell recruitment and augmenting the overall anti-tumor immune response (107). Concurrently, pembrolizumab reinforces this efficacy by abrogating tumor-mediated T-cell suppression, which collectively catalyzes a more robust immunological defense (108). These findings provide a compelling translational rationale for integrating chemokine axis blockade with Immune checkpoint inhibition (ICI). Additionally, the synergistic potential of CXCR4 antagonists, such as BL-8040, or CXCL12 inhibitors like NOX-A12, in combination with PD-1 inhibitors is currently under investigation to dismantle stroma-associated immunosuppressive barriers (108, 109). Nevertheless, the clinical translation of these chemokine-targeting strategies remains encumbered by certain limitations. Evidence supporting the navarixin-pembrolizumab regimen is primarily derived from Phase II trials in NSCLC. While these preliminary results underscore the immunomodulatory potential and clinical feasibility of the approach, they remain insufficient to confirm a consistent reduction in lung cancer incidence or a significant delay in disease progression.

#### 3.2.3 Multifunctional pathway-based combination strategies

At the direct interface of CAF-immune coupling, blocking both TGF-β and PD-L1 has a clear basis for changing the tumor microenvironment. TGF-β promotes CAF activation, myCAF maintenance, ECM deposition, T-cell exclusion, and EMT-related treatment tolerance. The PD-L1/PD-1 pathway directly limits effector T-cell function. Therefore, simultaneous blockade of TGF-β-driven stromal immunosuppression and PD-L1-mediated immune checkpoint signaling may enhance responses to immunotherapy. Bintrafusp alfa is a bifunctional fusion protein. It targets both PD-L1 and TGF-β. Early clinical and mechanistic studies showed that bintrafusp alfa had antitumor activity in some patients with NSCLC. These studies also showed that it could reduce TGF-β-induced EMT and immune-resistance phenotypes in experimental models (110, 111). However, subsequent phase III studies failed to demonstrate superiority of bintrafusp alfa over standard PD-1 blockade. In treatment-naive patients with advanced NSCLC and high PD-L1 expression, researchers compared bintrafusp alfa with pembrolizumab. Bintrafusp alfa did not show better efficacy (112). This result shows that dual-target strategies with strong biological support still need strict clinical testing. Several factors may explain this result. TGF-β signaling can have different effects in different settings. PD-L1 expression alone may not identify tumors driven by TGF-β or myCAF-related immune exclusion. Stromal pharmacodynamic biomarkers were also limited. The study also did not clearly separate TGF-β-dependent CAF states from TGF-β-independent CAF states. CAFs also have strong subtype diversity (113). Certain CAF populations may contribute to tissue homeostasis or support antitumor immune regulation. Using imaging mass cytometry across 1,070 NSCLC samples, Cords et al. identified 11 distinct CAF phenotypes and showed that CAF phenotype is a prognostic factor for patient survival. Tumor-like CAFs were associated with hypoxia, immune exhaustion and poor prognosis. Inflammatory CAFs and interferon-response CAFs were linked to an inflamed tumor microenvironment and improved survival. By contrast, increased matrix CAF density correlated with reduced immune infiltration and poorer survival (113).

Therefore, future TGF-β-targeting strategies should avoid non-selective pathway suppression. Instead, a more rational approach is biomarker-guided stromal modulation. LRRC15^+^ CAFs represent a candidate targetable state because they are enriched in lung cancer, associated with poor prognosis, and functionally linked to ECM deposition, M2-like macrophage polarization, and reduced CD8^+^ T-cell activity ^[68]^. LRRC15-restricted TGF-β trapping may limit systemic TGF-β blockade while concentrating stromal modulation within CAF-rich niches (68). Clinical development should prioritize patients with a spatially defined TGF-β-high, myCAF-rich, and immune-excluded phenotype, particularly those with low intratumoral CD8^+^ T-cell infiltration despite preserved stromal T-cell accumulation (69).

### 3.3 Theranostic strategies targeting the CAF-sEV communication axis

CAF-derived sEVs not only sustain pro-metastatic phenotypes but also offer strategic entry points for therapeutic intervention. Current approaches to modulating the CAF-sEV axis can be categorized into three distinct levels: the inhibition of sEV biogenesis and release, the blockade of cellular uptake, and the specific targeting of pathogenic molecular cargo. By intervening in the Ras-related protein Rab-27A/B (Rab27a/b) or Neutral sphingomyelinase 2 (nSMase2)/ceramide pathways, the export efficiency of deleterious CAF-derived payloads can be significantly attenuated, thereby undermining stromal-driven tumor invasion and immune evasion at their source (114). Furthermore, obstructing Heparan sulfate proteoglycan (HSPG)-dependent endocytosis serves to restrict the reception of these stroma-derived oncogenic signals by malignant cells (115). Additionally, the neutralization or molecular reprogramming of critical nucleic acid components, such as miR-210 or the Opa interacting protein 5-antisense RNA 1 (OIP5-AS1)/miR-142-5p/PD-L1 axis, facilitates a more selective attenuation of CAF-mediated metastasis and immunosuppression (93, 116). Although this field remains largely in the mechanistic research phase and faces substantial challenges regarding target selectivity, it holds significant translational promise for the precision theranostics of lung cancer (**Table 2**).

**Table 2:** Summary of potential CAF therapeutic strategies in lung cancer.

Category	Therapeutic target & mechanism	Representative agents	Current evidence & status	Limitations & challenges
ECM & Mechanical Barrier	Degrading HA to reduce IFP and alleviate mechanical load; enhancing drug and immune cell infiltration	PEGPH20, Pirfenidone, Nintedanib	Exploratory Phase II trials (e.g. PEGPH20 combined with PD-1); drug repurposing of anti-fibrotic agents	Risk of tumor exacerbation due to non-selective matrix depletion; limited benefit in unselected cohorts
Inflammatory Cytokine Network	Block IL-1β–IL-6–CRP axis to alleviate systemic inflammation and pro-tumor niche	Canakinumab (anti-IL-1β)	CANTOS suggested reduced lung cancer risk with canakinumab, but CANOPY-1 and CANOPY-2 showed no survival benefit in advanced NSCLC.	Limited transferability from prevention to treatment; lack of iCAF-based selection; inflammatory redundancy may weaken single IL-1β blockade.
Chemokine Axis & Immune Infiltration	Inhibit CXCR1/2 or CXCR4 to reduce immunosuppressive cell (TANs/MDSCs) recruitment	Navarixin, BL-8040, NOX-A12 (combined with PD-1 inhibitors)	Phase II studies showing improved CD8^+^ T cell infiltration and clinical feasibility	Evidence restricted to small cohorts; lack of robust long-term efficacy data
Multifunctional Immunostromal Targeting	Dual inhibition of PD-L1 and TGF-β to disrupt CAF-mediated immune exclusion	Bintrafusp alfa (M7824)	Bintrafusp alfa showed early activity, but phase III clinical failed to outperform pembrolizumab in PD-L1-high advanced NSCLC	PD-L1 alone may not identify TGF-β-driven immune exclusion; myCAF heterogeneity and limited stromal biomarkers remain key barriers
CAF-sEV Communication Axis	Inhibit sEV biogenesis (Rab27a/b), uptake (HSPG), or specific oncogenic cargo	miR-210, OIP5-AS1 (cargo); nSMase2 inhibitors	CAF-sEV cargos regulate EMT, immune escape and therapy resistance. No lung cancer CAF-sEV IND or Phase I program identified	Potential off-target effects on normal intercellular communication; lack of specific delivery systems

This table summarizes key therapeutic strategies that target CAF-mediated mechanisms in lung cancer. These strategies include ECM modelingremodelling, inflammatory cytokine blockade, chemokine-axis modulation, immunostromal co-targeting, and CAF-sEV intervention. The listed agents show the current progress of translational and clinical research. The evidence status reflects the maturity of each strategy. The limitations highlight the need for biomarker-guided patient selection and CAF subtype-specific therapeutic design.

CAF, cancer-associated fibroblast; CRP, C-reactive protein; CXCL8/12, C-X-C motif chemokine ligand 8/12; CXCR1/2/4, C-X-C motif chemokine receptor 1/2/4; ECM, extracellular matrix; HA, hyaluronic acid; hsCRP, high-sensitivity C-reactive protein; HSPG, heparan sulfate proteoglycan; IFP, interstitial fluid pressure; IL-1β/6, interleukin-1 beta/6; lncRNA, long non-coding RNA; miR, microRNA; NSCLC, non-small cell lung cancer; PBMC, peripheral blood mononuclear cell; PD-1, programmed cell death protein 1; PD-L1, programmed death-ligand 1; PTEN/PI3K/AKT, phosphatase and tensin homolog/phosphoinositide 3-kinase/protein kinase B; sEVs, small extracellular vesicles; TANs, tumor-associated neutrophils; TGF-β, transforming growth factor-beta; TKI, tyrosine kinase inhibitor.

### 3.4 Translational challenges and future perspectives of CAF-targeted therapy in lung cancer

CAF-targeted therapy in lung cancer has entered a new stage of development, yet clinical translation remains challenging. The key difficulty is that CAFs do not form a single, uniform therapeutic target. Single-cell and spatial studies in NSCLC have identified multiple CAF states, including matrix-associated, inflammatory, antigen-presenting, POSTN-positive, LRRC15-positive, and TGF-β-related subsets (30, 31, 43, 68, 69, 90, 113). Each CAF state shows distinct spatial distribution, immune interactions, and clinical associations. Therefore, broad CAF inhibition may have limited and inconsistent benefit. It may also impair fibroblast populations that support tissue protection or antitumor immunity.

This issue is particularly relevant to immune checkpoint blockade. Lung cancer studies show that specific CAF programs are associated with T-cell exclusion, ECM remodeling, and an immunosuppressive tumor microenvironment (30, 43, 69, 113). Matrix-associated CAFs can limit CD8^+^ T-cell access to tumor nests, while TGF-β-related fibroblast ecosystems are linked to resistance to immune checkpoint blockade (30, 69). In contrast, MHC-II^+^ antigen-presenting CAFs (apCAFs) may support local antitumor immunity in lung tumors (90). These findings indicate that CAF-directed therapy should not simply eliminate the stromal compartment. Instead, it should selectively suppress CAF states that promote immune exclusion and therapy resistance, while preserving CAF states that support immune activation or tissue homeostasis.

CAF plasticity further complicates therapeutic design. Most human NSCLC studies rely on single time-point samples. These studies define CAF heterogeneity but provide limited insight into how CAF states evolve during tumor progression or after therapy. POSTN^+^ CAFs and LRRC15^+^ CAFs are associated with ECM remodeling, immune suppression, and tumor progression in lung cancer (43, 68). However, it remains unclear whether these populations reflect stable fibroblast lineages or reversible activation programs shaped by tumor-derived signals, inflammation, and therapeutic pressure. This uncertainty limits the use of single CAF markers as treatment-selection biomarkers. Future studies should incorporate longitudinal tissue sampling before treatment, during early response, and at acquired resistance. They should also integrate spatial transcriptomics, multiplex imaging, and functional validation to determine whether CAF state transitions drive resistance or represent adaptive responses to therapy.

The mixed outcomes of CAF-related clinical strategies underscore the need for better patient selection. Anti-inflammatory therapy, HA-targeting approaches, chemokine-axis modulation, and dual PD-L1/TGF-β blockade have a clear biological rationale, but they have shown limited benefit in unselected NSCLC populations (103, 105, 106, 112). A likely explanation is that these strategies were not matched to the dominant stromal mechanism in each tumor. PD-L1 expression alone may not identify tumors with TGF-β-driven immune exclusion. Systemic inflammatory markers may also fail to capture tumors driven by inflammatory CAF signaling. Future trials should integrate stromal biomarkers with immune and spatial features. Relevant selection criteria may include TGF-β-high, myCAF-rich immune exclusion; LRRC15^+^ ECM-producing CAF enrichment; POSTN^+^ CAF accumulation; CXCL12-dominant stromal-myeloid interaction; and low intratumoral CD8^+^ T-cell infiltration despite stromal T-cell retention (30, 43, 68, 69, 113).

Future CAF-targeted therapy in lung cancer should shift from non-selective stromal inhibition to mechanism-guided stromal reprogramming. This approach requires validated CAF-state biomarkers and models that preserve tumor cells, fibroblasts, immune cells, and ECM architecture. Clinical studies should also incorporate pharmacodynamic endpoints, including changes in CAF states, ECM organization, CD8^+^ T-cell localization, myeloid infiltration, and circulating CAF-derived sEV signatures. These endpoints may clarify whether a treatment effectively remodels the tumor stroma. They may also help identify patients who are most likely to benefit from CAF-directed combinations therapies with immune checkpoint blockade, targeted therapy, or chemotherapy.

## 4 Conclusion

CAFs are key regulators of the lung cancer TME, characterized by profound heterogeneity and functional plasticity. By driving the continuous biosynthesis and structural reorganization of ECM components, CAFs activate integrin-mediated mechanotransduction pathways to construct a high-stiffness biophysical barrier associated with elevated interstitial hypertension. This mechanical landscape not only facilitates malignant cell invasion but also presents a formidable obstacle to the effective penetration and distribution of therapeutic agents. In the paracrine dimension, CAFs utilize an intricate secretome encompassing IL-6, TGF-β, HGF, and CXCL12 to reshape signaling networks in tumor cells, thereby inducing EMT and driving non-cell-autonomous drug resistance. Furthermore, CAF-mediated immune evasion operates through the dual modalities of physical sequestration and the recruitment of suppressive myeloid populations, thereby limiting responses to immune checkpoint blockade. Finally, sEVs serve as essential intercellular communication shuttles that further contribute to phenotypic reprogramming and the priming of distal pre-metastatic niches. Collectively, a deeper understanding of these multifaceted CAF-driven mechanisms will be instrumental in the development of next-generation precision therapies and stroma-targeted interventions for lung cancer.

Future stromal-targeted therapies will require closer integration of mechanistic studies, biomarker development, and rational combination strategies. Initial priorities should focus on the precise identification of pro-tumorigenic and tumor-suppressive CAF subpopulations through the integration of single-cell multi-omics and spatial transcriptomics, complemented by the profiling of circulating sEV signatures to facilitate patient stratification. Furthermore, therapeutic interventions should pivot toward stromal normalization, either by reversing pathogenic CAF phenotypes or employing anti-fibrotic agents to optimize the biophysical state of the microenvironment, thereby enhancing drug delivery efficiency. Beyond their utility as dynamic biomarkers for longitudinal monitoring, the inherent tissue tropism of CAF−sEVs presents a unique opportunity for the development of targeted delivery platforms. Clinical protocols must emphasize spatially-informed combinatorial strategies, employing the sequential or simultaneous blockade of pivotal axes, such as CXCL12/CXCR4 and TGF-β, to dismantle stroma-associated immunosuppressive barriers. A better understanding of the spatiotemporal dynamics of CAFs during lung cancer progression may facilitate the development of more effective stromal-targeted therapeutic strategies, moving from the singular targeting of malignant cells toward the holistic optimization of the tumor ecosystem.

## Glossary

α-SMA: Alpha-smooth muscle actin
AKT: Protein kinase B
apCAFs: Antigen-presenting cancer-associated fibroblasts
CAFs: Cancer-associated fibroblasts
CCL2: C-C motif chemokine ligand 2
CCR2: C-C motif chemokine receptor 2
c-MET: Mesenchymal-epithelial transition factor
COX-2: Cyclooxygenase-2
CRP: C-reactive protein
CXCL: C-X-C motif chemokine ligand (e.g. CXCL12, CXCL13)
CXCR: C-X-C motif chemokine receptor (e.g. CXCR2, CXCR4)
DNA: DeoxyriboNucleic Acid
ECM: Extracellular matrix
EGFR: Epidermal growth factor receptor
EMT: Epithelial-mesenchymal transition
ERK: Extracellular signal-regulated kinase
FAP-1: Fibroblast activation protein 1
FAs: Focal adhesions
FAK: Focal adhesion kinase
FN: Fibronectin
FN1: Fibronectin 1
HA: Hyaluronic acid
HGF: Hepatocyte growth factor
HSPG: Heparan sulfate proteoglycan
IARC: International Agency for Research on Cancer
ICB: Immune checkpoint blockade
ICI: Immune checkpoint inhibition
iCAFs: Inflammatory cancer-associated fibroblasts
IFN-γ: Interferon-gamma
IFP: Interstitial fluid pressure
IL: Interleukin (e.g. IL-1β, IL-6)
IL-6R: Interleukin-6 receptor
JAK: Janus kinase
JNK: c-Jun N-terminal kinase
lncRNAs: Long non-coding RNAs
LUAD: Lung Adenocarcinoma
LUSC: Lung Squamous Cell Carcinoma
MAPK: Mitogen-activated protein kinase
MDSCs: Myeloid-derived suppressor cells
miRNAs/miR: microRNAs
Nrf2: Nuclear factor erythroid 2-related factor 2
NSCLC: Non-small cell lung cancer
nSMase2: Neutral sphingomyelinase 2
OIP5-AS1: Opa interacting protein 5-antisense RNA 1
PBMC: Peripheral blood mononuclear cell
PD-1: Programmed cell death protein 1
PD-L1: Programmed death-ligand 1
PI3K: Phosphoinositide 3-kinase
PTEN: Phosphatase and tensin homolog
Rab27a/b: Ras-related protein Rab-27A/B
RhoA: Ras homolog family member A
ROCK: Rho-associated protein kinase
ROS: Reactive oxygen species
RTKs: Receptor tyrosine kinases
sEVs: Small extracellular vesicles
SMAD: Suppressor of mothers against decapentaplegic
Src: Proto-oncogene tyrosine-protein kinase Src
STAT3: Signal transducer and activator of transcription 3
TAMs: Tumor-associated macrophages
TAZ: Transcriptional coactivator with PDZ-binding motif
TGF-β: Transforming growth factor-beta
TGFBR1/2: Transforming growth factor-beta receptor ½
TKIs: Tyrosine kinase inhibitors
TME: Tumor microenvironment
Treg: Regulatory T-cell
UPF1: Up-frameshift suppressor 1
WHO: World Health Organization
YAP: Yes-associated protein
ZEB: Zinc finger E-box-binding homeobox.
